# Incidence and Independent Risk Factors for Obstetric Anal Sphincter Injuries: A Four-Year Retrospective Cohort Study from a UK Tertiary Maternity Unit

**DOI:** 10.3390/jcm15114396

**Published:** 2026-06-05

**Authors:** Maryada Malla, Stergios Doumouchtsis, Demetri Christian Panayi, Zainab Khan, Anand Singh

**Affiliations:** Epsom and St Helier University Hospitals NHS Trust, Wrythe Lane, Carshalton, Surrey SM5 1AA, UK; maryada.malla1@nhs.net (M.M.);

**Keywords:** obstetric anal sphincter injury, OASIS, episiotomy, forceps-assisted delivery, perineal trauma, risk factors

## Abstract

**Background:** Obstetric anal sphincter injuries (OASIS) are a major cause of long-term maternal morbidity. Identification of risk factors is central to prevention strategies. **Methods:** A retrospective cohort study was conducted including all singleton, term, cephalic vaginal births over a four-year period (September 2018 to September 2022) at a UK tertiary maternity unit. OASIS was defined as third- or fourth-degree perineal tears according to RCOG criteria. Multivariable logistic regression analysis was used to identify independent predictors. A pre-specified sensitivity analysis restricted to nulliparous women was performed. Secondary outcomes included postpartum haemorrhage (PPH), defined as blood loss >1 L. Reporting follows the STROBE statement for cohort studies. **Results:** Among 9586 vaginal births, 270 OASIS cases were identified, corresponding to an incidence of 2.82%. Independent predictors included nulliparity (aOR 7.12, 95% CI 5.07–10.01), Asian ethnicity (aOR 3.50, 95% CI 2.55–4.81), shoulder dystocia (aOR 3.45, 95% CI 1.89–6.32), and birthweight ≥4000 g (aOR 1.85, 95% CI 1.16–2.95). Maternal age ≥35 years showed a borderline association (aOR 1.34, 95% CI 0.99–1.80, *p* = 0.056). Using forceps as the reference, ventouse (aOR 0.28, 95% CI 0.17–0.47) and spontaneous vaginal delivery (aOR 0.23, 95% CI 0.13–0.39) were associated with lower OASIS odds. Episiotomy (recorded as a binary variable) was associated with lower adjusted odds of OASIS (aOR 0.27, 95% CI 0.17–0.44). PPH occurred in 21.5% of women with OASIS versus 6.5% without (*p* < 0.001). **Conclusions:** OASIS risk is driven by a combination of maternal, fetal, and intrapartum factors. Selective mediolateral episiotomy was associated with lower adjusted odds of OASIS in this cohort, but this is an observational finding and does not constitute proof of a causal protective effect. It should be interpreted cautiously given the retrospective design, the recording of episiotomy as a binary variable without procedural detail, and the substantial potential for residual confounding by indication. The findings support targeted perineal protection strategies and selective rather than routine episiotomy use.

## 1. Introduction

Obstetric anal sphincter injuries (OASIS), encompassing third- and fourth-degree perineal tears, represent the most severe form of perineal trauma during vaginal childbirth and are recognised as an important cause of postpartum morbidity [1]. Women who sustain OASIS are at increased risk of anal incontinence, faecal urgency, chronic perineal pain, dyspareunia, and long-term pelvic floor dysfunction; longitudinal follow-up studies indicate that a substantial proportion of affected women report persistent anal-incontinence symptoms years after delivery, with measurable effects on quality of life, sexual function, psychological wellbeing, and healthcare utilisation [1,2,3]. The cumulative and recurrent nature of sphincter injury across successive births further amplifies the long-term burden, making primary prevention a clinical priority [2].

Despite advances in intrapartum care, reported OASIS incidence in high-income settings commonly ranges between 1% and 7%, with variation driven by differences in case mix, parity distribution, operative vaginal delivery rates, diagnostic scrutiny, and local clinical practice [4,5,6,7,8,9,10]. Apparent increases in incidence in several countries over the past two decades are widely attributed to improved recognition and standardised classification rather than a true rise in injury, underscoring the influence of ascertainment on reported rates [8,9]. Large population-based and regional cohort studies consistently identify nulliparity, operative vaginal delivery—particularly forceps-assisted delivery—and fetal macrosomia as the dominant determinants of risk, while also highlighting contributions from Asian ethnicity, shoulder dystocia, advanced maternal age, prolonged second stage, and specific intrapartum interventions [4,5,6,7,8,9,10,11,12]. The magnitude of these associations varies between settings: for example, the adjusted risk attributable to nulliparity and to forceps delivery differs substantially across European and UK datasets, reflecting differences in obstetric practice and population structure [4,8,9,11].

At the same time, longitudinal registry and institutional data demonstrate that OASIS incidence can be modified through structured prevention strategies, including “hands-on” perineal support, controlled delivery of the fetal head, careful instrument selection, and context-specific use of mediolateral or lateral episiotomy [4,5,8,9,10,13,14,15,16]. Routine episiotomy is no longer recommended, as it does not reduce severe perineal trauma and may increase morbidity, whereas selective mediolateral episiotomy appears protective in high-risk operative vaginal births, particularly among nulliparous women [4,8,13,14,15,16,17]. However, the evidence base for episiotomy is complicated by confounding by indication—episiotomy is preferentially performed in births already judged to be at higher risk—which limits the conclusions that can be drawn from observational data and has produced apparently contradictory findings across studies [13,14,15,16,17].

In the United Kingdom, the introduction of the national OASI Care Bundle and successive RCOG Green-top guidance has changed perineal protection practice over the past decade, and current RCOG guidance advocates selective rather than routine episiotomy alongside active perineal protection [1,18]. Despite this, contemporary UK data that integrate maternal, fetal and intrapartum factors within a single multivariable framework, and that reflect current tertiary-unit practice in an ethnically diverse population in the post-Care-Bundle era, remain limited; much of the existing UK evidence predates these practice changes or is derived from national administrative datasets that lack granular intrapartum detail [9,18]. Unit-level cohort studies with contemporaneous data are therefore needed to inform locally relevant risk stratification and to contextualise national prevention strategies.

This study aims to determine the incidence of OASIS and to identify independent risk factors in a large, recent UK tertiary-unit cohort, with particular focus on mode of delivery and the role of selective mediolateral episiotomy in mitigating severe perineal trauma. By integrating maternal, fetal and intrapartum variables within a single multivariable framework, applying formal model diagnostics, and reporting findings using STROBE-compliant methodology, the study provides contemporary, ethnically diverse, unit-level evidence intended to inform selective perineal protection strategies in current UK practice and to complement national administrative analyses.

## 2. Materials and Methods

### 2.1. Study Design and Setting

This retrospective cohort study was conducted at a maternity unit located in South-West London, United Kingdom. The unit is part of a teaching hospital within the National Health Service (NHS) and serves a diverse urban population encompassing a wide range of ethnic and socioeconomic backgrounds. The maternity service manages approximately 4000 deliveries annually and is well equipped to care for both low- and high-risk pregnancies. It includes dedicated spaces for labour ward care, a midwifery-led birth centre, and home birth services. The institution also functions as a referral centre for complex obstetric cases within the region.

### 2.2. Study Population

The study included all deliveries that occurred in the hospital during a four-year period from September 2018 to September 2022, using the institutional delivery registry.

**Figure 1:**
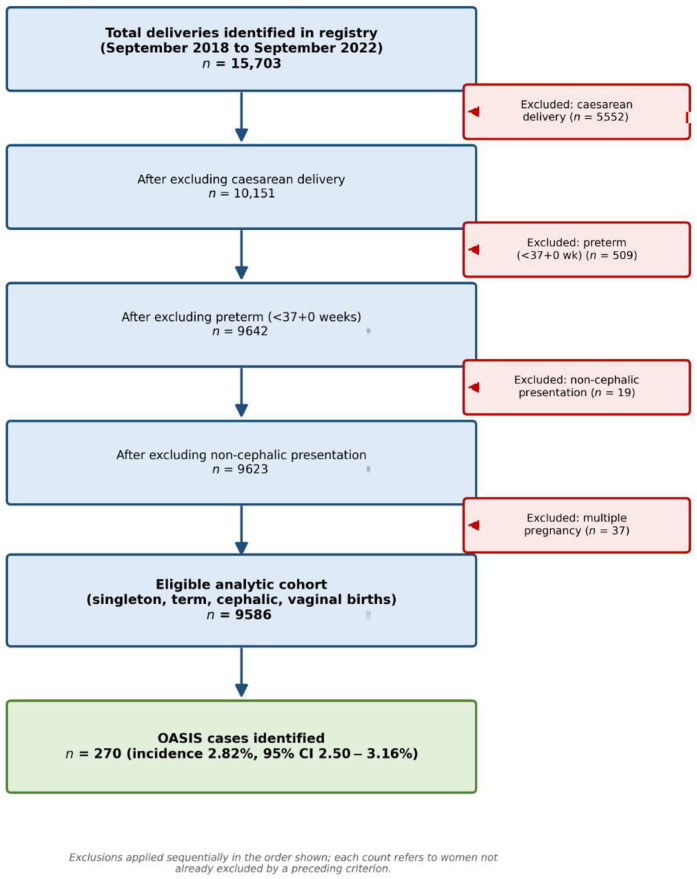
STROBE flow diagram of cohort selection.

Exclusion criteria included caesarean delivery, preterm birth, non-cephalic presentation, and multiple pregnancy. A census sampling approach was employed to include the entire population of eligible deliveries during the study period. The flow of deliveries from the total registry to the analytic cohort, including reasons for exclusion, is shown in Figure 1 (STROBE flow diagram).

### 2.3. Outcome Definition

The primary outcome was the occurrence of obstetric anal sphincter injuries (OASIS), defined as third- or fourth-degree perineal tears sustained during vaginal delivery. Classification followed the Royal College of Obstetricians and Gynaecologists (RCOG) Green-top Guideline No. 29 [1], consistent with the International Continence Society/International Urogynecological Association joint terminology for obstetric pelvic floor disorders [19], which specifies:

- **Grade 3a:** Partial tear of the external anal sphincter (EAS) involving less than 50% of its thickness.
- **Grade 3b:** More than 50% thickness of the EAS torn.
- **Grade 3c:** Both the EAS and the internal anal sphincter (IAS) torn.
- **Grade 4:** Disruption of EAS, IAS, and the anorectal mucosa.

All diagnoses were confirmed by attending obstetricians at the time of delivery and documented in operative or delivery notes. Where grading was performed by resident doctors or midwives, final verification was made by a supervising consultant.

Secondary outcome: Postpartum haemorrhage (PPH) was defined as estimated blood loss greater than 1 L following delivery, as recorded in the delivery registry. The association between OASIS and PPH was assessed as a secondary outcome.

### 2.4. Data Collection

Data were obtained from the unit’s electronic maternity information system, in which clinical and demographic details are recorded contemporaneously by the attending midwifery and obstetric staff at the time of admission, labour, and delivery as part of routine care. A structured electronic data extract covering all registerable deliveries during the study period was generated by the Trust’s clinical audit and information team using predefined data fields and was provided to the study team in tabular form. The extract was then pseudonymised, with direct patient identifiers (hospital number and date of birth) removed and maternal age derived prior to analysis; no free-text clinical notes were accessed.

The following variables were extracted for each delivery. Maternal factors: maternal age (years, derived), booking body mass index (BMI, kg/m^2^), parity, pre-existing or gestational diabetes (as coded in the maternity record), and ethnicity (recorded using the National Health Service maternity dataset ethnic-category classification as documented in the maternity information system). Fetal and neonatal factors: birthweight (grams), infant sex, shoulder dystocia (recorded by the attending clinician as present or absent), and gestational age at birth (completed weeks). Intrapartum factors: mode of delivery, onset of labour, episiotomy, and the professional role of the person conducting the delivery. Operative vaginal delivery was defined as instrumental vaginal birth using forceps or vacuum (ventouse); the maternity system additionally records the operative-delivery subtype (low-cavity, mid-cavity or rotational forceps; ventouse with or without rotation), which was used for the sensitivity analysis described in Section 2.5. Episiotomy was recorded as a binary variable (yes/no); in this unit episiotomy is performed using the mediolateral technique, but the angle, indication, and timing of the incision are not recorded in the electronic system. Onset of labour was categorised as spontaneous or induced. Birthweight was analysed as a continuous variable, with macrosomia pre-defined as birthweight ≥4000 g, and maternal age was additionally dichotomised at ≥35 years for the primary model. The primary outcome (OASIS) and the secondary outcome (postpartum haemorrhage) were defined as described in Section 2.3.

Several clinically relevant variables were not reliably captured within the registry and could therefore not be analysed, namely previous OASIS, duration of second stage of labour, epidural analgesia in usable form, fetal head position at delivery for instrumental births, and detailed information on induction or augmentation regimens. The implications of these omissions are discussed in Section 5.

### 2.5. Statistical Analysis

As a census approach was used (every eligible delivery during the inclusion window), no formal sample-size calculation was performed; the resulting cohort and event count were sufficient to support multivariable logistic regression with the planned covariate set.

Descriptive statistics were used to summarise cohort characteristics. The normality of continuous variables was assessed visually using histograms and Q–Q plots and confirmed using the Shapiro–Wilk test; normally distributed variables are summarised as mean (SD) and non-normal variables as median (IQR). Univariable comparisons between women with and without OASIS were performed using chi-square or Fisher’s exact tests for categorical variables and *t*-tests or Mann–Whitney U tests for continuous variables, as appropriate.

Multivariable logistic regression was used to identify independent predictors of OASIS, adjusting for clinically relevant covariates selected *a priori* based on the existing literature: parity, maternal age (≥35 vs. <35 years), BMI (≥30 vs. <30 kg/m^2^), ethnicity, shoulder dystocia, mode of delivery (spontaneous vaginal, ventouse, forceps; forceps as reference), episiotomy, birthweight (≥4000 vs. <4000 g), and onset of labour. All covariates were entered simultaneously rather than via stepwise selection. Adjusted odds ratios (aORs) with 95% confidence intervals (CIs) were reported. Statistical significance was set at *p* < 0.05.

Multicollinearity was assessed using variance inflation factors (VIFs); the highest observed VIF was 3.22, indicating no problematic collinearity. Model discrimination was assessed using the area under the receiver operating characteristic curve (C-statistic = 0.778), and calibration was assessed using the Hosmer–Lemeshow goodness-of-fit test (χ^2^ = 12.59, df = 8, *p* = 0.127).

A pre-specified sensitivity analysis restricted to nulliparous women was performed to evaluate the robustness of the findings in this higher-risk subgroup (Supplementary Table S1a). An additional sensitivity analysis was performed with maternal age and birthweight modelled as continuous rather than dichotomised variables (Supplementary Table S1b). Missing data were minimal (<8% for all variables; per-variable missingness reported in Supplementary Table S2) and were handled using complete-case analysis. Analyses were performed using R version 4.3.1.

### 2.6. Use of Artificial Intelligence Tools

AI-based writing-assistance tools were used solely to support language editing and structural refinement of selected sections of the manuscript draft, with all content reviewed and approved by the authors, who accept full responsibility for the integrity of the work. A corresponding statement is provided in the Acknowledgments.

### 2.7. Ethical Considerations

This study used routinely collected, retrospectively extracted maternity registry data and was conducted as part of the unit’s clinical service-evaluation and quality-improvement programme. The project was registered with the Epsom and St Helier University Hospitals NHS Trust Clinical Audit Department on 18 September 2024 under approved audit reference number 2425WC018. In line with the NHS Health Research Authority decision tool, formal Research Ethics Committee approval was not required, as the work constituted service evaluation using anonymised data with no change to patient management. All data were de-identified prior to analysis. The study was conducted in accordance with the Declaration of Helsinki.

## 3. Results

Of the 15,703 deliveries during the study period, 9586 term, singleton, cephalic, vaginal births met the study inclusion criteria after sequential exclusion of caesarean delivery (*n* = 5552; 10,151 remaining), preterm birth (*n* = 509; 9642 remaining), non-cephalic presentation (*n* = 19; 9623 remaining), and multiple pregnancy (*n* = 37; 9586 remaining), as shown in the STROBE flow diagram (Figure 1). Obstetric anal sphincter injury (OASIS), defined as third- and fourth-degree perineal tears, occurred in 270 women, corresponding to an overall incidence of 2.82% among eligible vaginal births. Most cases were third-degree tears.

Grade 3a tears accounted for 143 cases (53.0%), followed by grade 3b tears in 95 cases (35.2%) and grade 3c tears in 24 cases (8.9%). Fourth-degree tears were uncommon, occurring in eight women (3.0%).

The mean maternal age of the cohort was 32.3 ± 4.9 years. The mean booking body mass index (BMI) was 25.8 ± 5.3 kg/m^2^, and the mean birthweight was 3375 ± 437 g. The cohort was ethnically diverse, with the majority of women recorded as British, followed by Asian, White-Other, Black, and other minority ethnic groups. Diabetes, as recorded in the dataset, was present in approximately 10.4% of the cohort.

Table 1 summarises demographic, intrapartum, and neonatal characteristics according to obstetric anal sphincter injury status. Women sustaining OASIS were more likely to be nulliparous and the distribution of ethnicity differed significantly between groups (χ^2^ test, *p* < 0.001), with women of Asian ethnicity comprising a higher proportion of OASIS cases. Crude OASIS rates differed significantly by mode of delivery (χ^2^ test *p* < 0.001). Forceps-assisted delivery was associated with the highest rate, compared with ventouse and spontaneous vaginal delivery. Shoulder dystocia was also more frequent among women with OASIS. Episiotomy was more frequently performed among women who sustained OASIS in crude analyses (35.6% vs. 27.7%).

Among the neonatal factors, the sex of the neonate was not significantly associated with OASIS in this analysis.

Additional intrapartum and care-related characteristics (Table 2) demonstrated significant differences between groups. Spontaneous onset of labour was more frequent among women with OASIS, whereas history of a previous caesarean section did not differ significantly between groups. Differences were also observed in intrapartum care characteristics, including the professional role of the individual conducting the delivery.

In the multivariable logistic regression analysis, nulliparity was the strongest independent predictor of obstetric anal sphincter injury (OASIS), with an adjusted odds ratio (aOR) of 7.12 (95% CI 5.07–10.01, *p* < 0.001). Asian ethnicity was also independently associated with increased odds of OASIS (aOR 3.50, 95% CI 2.55–4.81, *p* < 0.001), as were shoulder dystocia (aOR 3.45, 95% CI 1.89–6.32, *p* < 0.001) and delivery of a macrosomic infant (birthweight ≥4000 g; aOR 1.85, 95% CI 1.16–2.95, *p* = 0.009). Advanced maternal age (≥35 years) showed a borderline association in the primary model (aOR 1.34, 95% CI 0.99–1.80, *p* = 0.056). Adjusted estimates for the remaining ethnicity categories are presented in Table 3.

**Figure 2:**
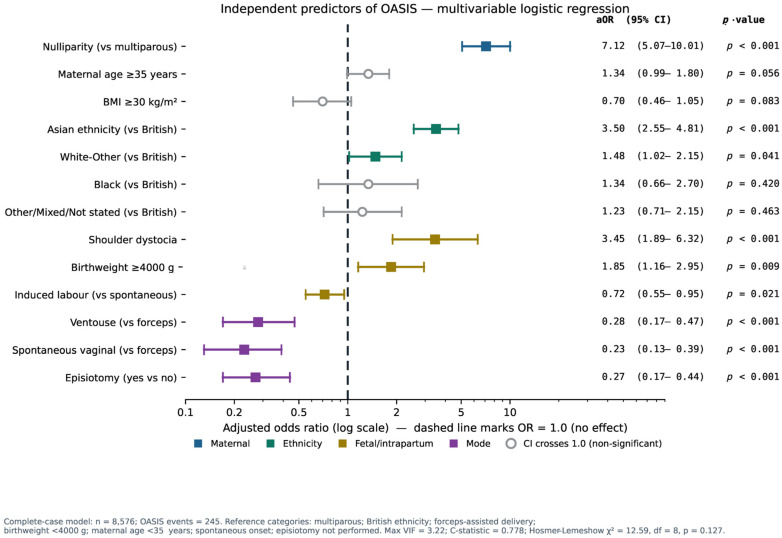
Forest plot: multivariable logistic regression for OASIS.

Figure 2 presents a forest plot of the multivariable logistic regression model, displaying the adjusted odds ratios and 95% confidence intervals for all independent predictors of OASIS.

Using forceps-assisted delivery as the reference category, both ventouse-assisted delivery (aOR 0.28, 95% CI 0.17–0.47, *p* < 0.001) and spontaneous vaginal delivery (aOR 0.23, 95% CI 0.13–0.39, *p* < 0.001) were associated with lower odds of OASIS. Mediolateral episiotomy was associated with lower adjusted odds of OASIS (aOR 0.27, 95% CI 0.17–0.44, *p* < 0.001). An interaction between forceps-assisted delivery and episiotomy was explored in the multivariable model; however, no statistically significant interaction was observed. A pre-specified sensitivity analysis restricted to nulliparous women demonstrated effect estimates of similar direction and magnitude (Supplementary Table S1a). A further exploratory analysis stratifying operative vaginal delivery into detailed subtypes (low-cavity, mid-cavity and rotational forceps; ventouse with and without rotation) is presented in Supplementary Table S1c; mid-cavity forceps showed a borderline elevation in odds compared with low-cavity forceps (aOR 1.90, 95% CI 0.99–3.65, *p* = 0.055), while the rotational forceps subgroup was too small (*n* = 36, 3 OASIS events) to yield precise estimates.

All registerable deliveries during September 2018 to September 2022: *n* = 15,703. Exclusions were applied sequentially in the order shown: caesarean delivery (*n* = 5552; 10,151 remaining); preterm birth <37 + 0 weeks (*n* = 509; 9642 remaining); non-cephalic presentation (*n* = 19; 9623 remaining); and multiple pregnancy (*n* = 37; 9586 remaining). Each count refers to deliveries not already excluded by a preceding criterion. The eligible analytic cohort comprised *n* = 9586 singleton, term, cephalic, vaginal births. OASIS (third- or fourth-degree tears) was identified in *n* = 270, corresponding to an incidence of 2.82% (95% CI 2.50–3.16%).

The forest plot demonstrates adjusted odds ratios for factors associated with obstetric anal sphincter injury, showing increased risk with nulliparity, forceps delivery, and increasing birthweight, while episiotomy is associated with lower odds of OASIS after adjustment. Estimates derive from a complete-case multivariable logistic regression (*n* = 8576; OASIS events = 245). Reference categories: multiparous; British ethnicity; forceps-assisted delivery; birthweight <4000 g; maternal age <35 years; spontaneous onset; and episiotomy not performed.

As a secondary outcome, women who sustained OASIS experienced significantly higher rates of postpartum haemorrhage. Blood loss exceeding 1 L occurred in 21.5% of women with OASIS compared with 6.5% of those without OASIS (*p* < 0.001), highlighting the broader maternal morbidity burden associated with severe perineal trauma. This crude association is reported descriptively; PPH was not modelled as an independent multivariable outcome in the present analysis.

## 4. Discussion

### 4.1. Principal Findings

In this large contemporary UK cohort, the incidence of obstetric anal sphincter injury (OASIS) was 2.82% among term singleton cephalic vaginal births, consistent with rates reported in recent European population-based and regional studies (2–4%) [4,5,6,8,9,10,16]. Several maternal, intrapartum, and neonatal factors were associated with OASIS in crude analyses. Nulliparity, Asian ethnicity, forceps-assisted delivery, shoulder dystocia, and neonatal macrosomia were more frequent among women sustaining OASIS. In contrast, ventouse-assisted delivery and infant sex were not significantly associated with OASIS in unadjusted analyses.

Importantly, episiotomy was more commonly performed in births complicated by OASIS in crude analyses, yet showed an inverse association after adjustment for confounders in multivariable modelling. This unadjusted–adjusted reversal is consistent with confounding by indication, in which episiotomy is preferentially performed in clinical contexts judged to confer higher risk; the implications for clinical interpretation are discussed below.

### 4.2. Comparison with Existing Literature

The observed incidence is similar to that reported from national datasets in Austria, Norway and England, indicating that practice and risk profiles in this UK tertiary unit broadly reflect those in other high-income settings. Severe perineal trauma therefore remains a common and clinically important complication of vaginal birth even in well-resourced maternity services and underscores the need for continued prevention strategies despite advances in intrapartum care [4,5,6,8,9,10,16].

Nulliparity was the strongest independent predictor of OASIS in this study, conferring a substantial increase in adjusted risk compared with multiparity. This finding is highly consistent with large registry and cohort analyses in which primiparity repeatedly emerges as the dominant non-modifiable risk factor for anal sphincter injury [4,5,6,8,9,10,11,16].

Asian ethnicity was also independently associated with OASIS in this cohort, aligning with prior UK and international work demonstrating higher rates of severe perineal trauma among women of South Asian background [7,9]. The contributors to this association are likely to be multifactorial. Possible explanations include differences in pelvic dimensions and fetal–maternal proportionality, variations in connective tissue properties, differences in body composition, and culturally or socioeconomically patterned differences in care-seeking and access to antenatal care. Practice-related factors, such as variation in the application of perineal protection manoeuvres or thresholds for episiotomy, may also play a role. Speculative single-mechanism explanations should be avoided, and these contributors warrant further qualitative and observational research.

Fetal macrosomia and shoulder dystocia were also associated with increased OASIS risk, consistent with the mechanical stresses imposed on the perineum during difficult births. Registry data from Norway and England demonstrate stepwise increases in severe perineal trauma with increasing birthweight, and several studies identify shoulder dystocia as a particularly high-risk context in which rotational or traction manoeuvres may concentrate force at the sphincter complex [4,5,6,8,9,10,12,16]. In our cohort, BMI ≥ 30 kg/m^2^ was not significantly associated with increased OASIS risk and the point estimate was directed toward lower odds (aOR 0.70, 95% CI 0.46–1.05), consistent with a prior UK study reporting that higher maternal BMI was associated with reduced incidence of perineal trauma overall [20].

Instrumental delivery was another important determinant of OASIS in this cohort. Forceps-assisted delivery has consistently been associated with higher rates of OASIS compared with vacuum delivery in observational studies [21]. This pattern mirrors findings from large observational studies and systematic reviews demonstrating substantially higher OASIS risk with forceps compared with spontaneous vaginal birth, with vacuum delivery occupying an intermediate position [4,5,8,9,10,16,17]. The exploratory subtype analysis (Supplementary Table S1c) further suggests that within the forceps group, mid-cavity forceps were associated with higher odds of OASIS than low-cavity (outlet) forceps, consistent with prior work from this institution and others indicating that rotational and mid-cavity instruments concentrate greater perineal force than outlet instruments [21]. The rotational forceps subgroup was small (*n* = 36) and the corresponding estimate imprecise.

Heterogeneity in outcome definitions and reporting across studies further underscores the importance of developing standardised core outcome sets in perineal trauma research, as highlighted by the CHORUS international collaboration [22].

### 4.3. Clinical Implications

In this cohort, episiotomy was associated with lower adjusted odds of OASIS after multivariable adjustment, although crude rates were higher in women undergoing episiotomy. This unadjusted–adjusted reversal indicates that episiotomy is preferentially used in higher-risk situations and adjusts only partially for that confounding by indication. The adjusted estimate should therefore not be interpreted as a causal protective effect.

These findings align with evidence suggesting that mediolateral episiotomy may reduce OASIS risk during operative vaginal delivery, particularly in nulliparous women. However, episiotomy was recorded as a binary variable in this dataset, and information on incision angle, indication, timing relative to crowning, side, or operator seniority was not available. Therefore, it cannot be confirmed that episiotomies were performed at the recommended 45–60° angle, which may influence the observed association. Residual confounding from variables not captured in the registry—particularly previous OASIS, second-stage duration, epidural analgesia, fetal head position, and induction or augmentation regimens—cannot be excluded.

The results of this study also resonate with broader European experience of structured perineal protection programmes. Studies from Norway have demonstrated that multifaceted interventions including hands-on perineal support, controlled delivery of the fetal head, correct mediolateral episiotomy technique, and focused staff training can significantly reduce OASIS incidence. Similar national analyses from France highlight that restrictive episiotomy policies can be implemented safely when accompanied by careful monitoring and attention to technique.

Taken together, these findings emphasise that OASIS risk reflects a complex interplay of maternal, fetal and intrapartum factors. In clinical practice, combinations such as nulliparity, Asian ethnicity, fetal macrosomia, shoulder dystocia and operative vaginal delivery identify women at particularly high risk. Enhanced perineal support, careful selection of operative instruments and selective mediolateral episiotomy may therefore be important components of prevention strategies in these scenarios [1,2,4,5,6,7,8,9,10,14,15,16,17].

These findings are also broadly consistent with national prevention strategies such as the OASI Care Bundle, which combines improved perineal support techniques, selective mediolateral episiotomy use and staff training to reduce obstetric anal sphincter injuries [18].

The substantially elevated rate of postpartum haemorrhage among women sustaining OASIS (21.5% vs. 6.5%) underscores the cumulative maternal morbidity associated with severe perineal trauma and reinforces the importance of anticipatory haemorrhage management in high-risk births.

## 5. Strengths and Limitations

Strengths of this study include the large contemporary cohort, use of standardised RCOG definitions, and multivariable analysis adjusting for key confounders, with formal assessment of multicollinearity, model discrimination, and calibration. The single-centre design enables coherent application of unit protocols and consistent diagnostic standards across the inclusion period. Reporting follows the STROBE statement, with a documented sequential cohort-selection process (Figure 1) and per-variable missingness reported in the Supplementary Material.

Limitations include the retrospective design, potential documentation bias, lack of data on episiotomy angle, and absence of long-term functional outcomes such as anal continence. Residual confounding cannot be excluded, particularly for intrapartum interventions such as episiotomy that are influenced by clinician judgement and delivery circumstances. Information on previous obstetric anal sphincter injury was not available and could not be included in the analysis; as previous OASIS is a recognised risk factor for recurrence and previous mode of delivery more broadly has been shown to influence perineal trauma risk in subsequent births [23], this represents an important limitation. The registry also did not reliably capture duration of second stage, epidural analgesia, or detailed information on induction or augmentation regimens—variables that have been associated with severe perineal trauma in other studies. Fetal head position at delivery was documented only for spontaneous vaginal births (as occipito-anterior versus ‘abnormal presentation’) and was not recorded for instrumental deliveries; this is the population in which malposition is most clinically relevant and therefore represents an important limitation. For the primary multivariable model, operative vaginal delivery was collapsed into single forceps and ventouse categories for parsimony; an exploratory analysis stratifying by detailed mode (low-cavity, mid-cavity and rotational forceps; ventouse with and without rotation) is presented in Supplementary Table S1c, but some subtype subgroups (in particular rotational forceps, *n* = 36) were too small for precise estimates. The secondary outcome (PPH) was assessed descriptively rather than within a multivariable framework. Further prospective research is needed to optimise targeted OASIS prevention strategies, particularly among nulliparous women undergoing operative vaginal delivery.

## 6. Conclusions

OASIS remains a significant complication of vaginal birth, particularly among nulliparous women, those of Asian ethnicity, and in the context of macrosomia, shoulder dystocia, and forceps-assisted delivery. Selective mediolateral episiotomy was associated with lower adjusted odds of OASIS, but this finding should be interpreted cautiously given the retrospective design, the recording of episiotomy as a binary variable without procedural detail, and the substantial potential for residual confounding by indication. These findings support refined intrapartum risk stratification and selective rather than routine perineal protection strategies. Women sustaining OASIS also experienced substantially higher rates of postpartum haemorrhage, emphasising the broader maternal morbidity associated with this injury.

## Figures and Tables

**Table 1:** Baseline maternal, fetal and intrapartum characteristics by OASIS status.

Variable	No OASIS ( *n* = 9316)	OASIS ( *n* = 270)	*p* -Value
**Demographic variables**			
Maternal age ≥ 35 years	3023/9316 (32.4%)	78/270 (28.9%)	0.243
BMI ≥ 30 kg/m^2^	1654/9041 (18.3%)	32/261 (12.3%)	0.016
Nulliparity	3782/9316 (40.6%)	208/270 (77.0%)	<0.001
Diabetes (any)	981/9316 (10.5%)	17/270 (6.3%)	0.032
**Ethnicity**			**<0.001**
British	5399/9316 (58.0%)	121/270 (44.8%)	
Asian *	1386/9316 (14.9%)	81/270 (30.0%)	
White-Other	1410/9316 (15.1%)	43/270 (15.9%)	
Black †	470/9316 (5.0%)	10/270 (3.7%)	
Other/Mixed/Not stated	651/9316 (7.0%)	15/270 (5.6%)	
**Delivery-related variables**			
Shoulder dystocia	170/9300 (1.8%)	16/270 (5.9%)	<0.001
**Mode of delivery**			**<0.001**
Spontaneous vaginal	7516/9316 (80.7%)	185/270 (68.5%)	
Forceps-assisted	789/9316 (8.5%)	61/270 (22.6%)	
Ventouse	1011/9316 (10.9%)	24/270 (8.9%)	
Episiotomy (yes)	2386/8617 (27.7%)	90/253 (35.6%)	0.007
**Neonatal variables**			
Birthweight ≥ 4000 g	736/9312 (7.9%)	27/270 (10.0%)	0.254
Male infant	4651/9312 (49.9%)	144/270 (53.3%)	0.300

* Asian ethnicity includes Indian, Pakistani, Bangladeshi, and Asian-Other. † Black ethnicity includes Black African, Black Caribbean, and Black-Other. Denominators vary slightly because of missing data for some variables in the delivery registry. Per-variable missingness is reported in Supplementary Table S2.

**Table 2:** Additional intrapartum care and obstetric factors by OASIS status.

Variable	No OASIS ( *n* = 9316)	OASIS ( *n* = 270)	*p* -Value
**Labour characteristics**			
Spontaneous onset of labour	5363/9291 (57.7%)	174/270 (64.4%)	0.032
Induced labour	3928/9291 (42.3%)	96/270 (35.6%)	
**Previous obstetric history**			
Previous caesarean section (≥1)	377/9316 (4.0%)	16/270 (5.9%)	0.168
**Intrapartum care factors**			
Same midwife throughout labour	8028/9081 (88.4%)	233/264 (88.3%)	1.000
**Person conducting delivery**			**0.001**
Midwife-led delivery	5000/9179 (54.5%)	124/264 (47.0%)	
Hospital doctor present	1855/9179 (20.2%)	80/264 (30.3%)	
Student midwife involvement	2201/9179 (24.0%)	57/264 (21.6%)	
Other	123/9179 (1.3%)	3/264 (1.1%)	

Denominators vary slightly because of missing data for some variables in the delivery registry. Per-variable missingness is reported in Supplementary Table S2.

**Table 3:** Independent predictors of OASIS—multivariable logistic regression.

Predictor	Adjusted OR	95% CI	*p* -Value
Nulliparity	7.12	5.07–10.01	<0.001
Asian ethnicity	3.50	2.55–4.81	<0.001
Shoulder dystocia	3.45	1.89–6.32	<0.001
Birthweight ≥ 4000 g	1.85	1.16–2.95	0.009
White-Other ethnicity (vs British)	1.48	1.02–2.15	0.041
Maternal age ≥ 35 years	1.34	0.99–1.80	0.056
Black ethnicity (vs British)	1.34	0.66–2.70	0.420
Other/Mixed/Not stated (vs British)	1.23	0.71–2.15	0.463
BMI ≥ 30 kg/m^2^	0.70	0.46–1.05	0.083
Induced labour (vs spontaneous)	0.72	0.55–0.95	0.021
Ventouse vs. forceps	0.28	0.17–0.47	<0.001
Spontaneous vs. forceps	0.23	0.13–0.39	<0.001
Episiotomy (yes vs. no)	0.27	0.17–0.44	<0.001

Reference categories: multiparous; British ethnicity; forceps-assisted delivery; birthweight < 4000 g; maternal age < 35 years; spontaneous onset; episiotomy not performed. Complete-case analysis *n* = 8576; OASIS events = 245. Multicollinearity excluded (max VIF = 3.22). Discrimination C-statistic = 0.778; Hosmer–Lemeshow χ^2^ = 12.59, df = 8, *p* = 0.127.

## Data Availability

The anonymised aggregate data underlying the findings of this study are available from the corresponding author on reasonable request, subject to approval from the Epsom and St Helier University Hospitals NHS Trust Information Governance team. Individual-level patient data cannot be shared owing to NHS data-protection requirements.

## Supplementary Materials

The following supporting information can be downloaded at: https://www.mdpi.com/article/10.3390/jcm15114396/s1. Supplementary Table S1: (a)—pre-specified sensitivity analysis restricted to nulliparous women; (b)—sensitivity analysis with maternal age and birthweight modelled as continuous variables; (c)—exploratory analysis stratifying operative vaginal delivery by detailed mode (low-cavity, mid-cavity and rotational forceps; ventouse with and without rotation); Supplementary Table S2—per-variable missingness across the analytic cohort.
