# Supplementary material for: Incidence and Independent Risk Factors for Obstetric Anal Sphincter Injuries: A Four-Year Retrospective Cohort Study from a UK Tertiary Maternity Unit
Source: J Clin Med. 2026 Jun 5;15(11):4396. doi: 10.3390/jcm15114396 (PMC13257661; doi:10.3390/jcm15114396)
Supplement: Supplementary file 1 [file jcm-15-04396-s001.zip › jcm-4283858-supplementary.pdf]

Supplementary Table S1a. Sensitivity analysis: Nulliparous women only

Predictor	aOR	95% CI	p-value
Spontaneous vaginal vs forceps	0.23	0.13–0.41	<0.001
Ventouse vs forceps	0.29	0.17–0.49	<0.001
Episiotomy (yes vs no)	0.27	0.16–0.45	<0.001
Shoulder dystocia	2.01	0.86–4.71	0.108
Asian ethnicity (vs British)	3.42	2.37–4.92	<0.001
White-Other (vs British)	1.42	0.92–2.18	0.111
Black (vs British)	1.50	0.67–3.36	0.325
Other/Mixed/Not stated (vs British)	1.26	0.67–2.36	0.470
Maternal age ≥35 years	1.36	0.95–1.94	0.091
BMI ≥30 kg/m²	0.76	0.48–1.20	0.235
Birthweight ≥4,000 g	1.54	0.82–2.91	0.181
Induced labour (vs spontaneous)	0.74	0.54–1.01	0.059

Complete-case multivariable logistic regression in nulliparous women only.

n = 3,593; OASIS events = 189.

Reference categories: forceps-assisted delivery; British ethnicity; episiotomy not performed; birthweight <4,000 g; maternal age <35 years; BMI <30 kg/m²; spontaneous onset.

Supplementary Table S1b. Sensitivity analysis: Continuous age and birthweight

Predictor	aOR	95% CI	p-value
Nulliparity (vs multiparous)	8.12	5.73–11.51	<0.001
Maternal age (per year)	1.04	1.01–1.07	0.006
Birthweight (per kg)	1.84	1.33–2.54	<0.001
Asian ethnicity (vs British)	3.83	2.76–5.32	<0.001
White-Other (vs British)	1.43	0.98–2.08	0.061
Black (vs British)	1.46	0.72–2.97	0.290
Other/Mixed/Not stated (vs British)	1.29	0.74–2.26	0.368
Shoulder dystocia	3.40	1.87–6.19	<0.001
Spontaneous vaginal vs forceps	0.24	0.14–0.42	<0.001
Ventouse vs forceps	0.29	0.18–0.49	<0.001
Episiotomy (yes vs no)	0.26	0.16–0.42	<0.001
BMI ≥30 kg/m²	0.68	0.45–1.03	0.067
Induced labour (vs spontaneous)	0.77	0.59–1.02	0.066

Complete-case multivariable logistic regression with maternal age and birthweight modelled as continuous variables.

n = 8,576; OASIS events = 245.

Reference categories: multiparous; forceps-assisted delivery; British ethnicity; episiotomy not performed; BMI <30 kg/m²; spontaneous onset.

Supplementary Table S1c. Exploratory sensitivity analysis — detailed mode of operative vaginal delivery.

Predictor	aOR	95% CI	p-value
Nulliparity (vs multiparous)	7.11	5.05–9.99	<0.001
Asian ethnicity (vs British)	3.47	2.53–4.77	<0.001
White-Other (vs British)	1.46	1.00–2.12	0.048
Black (vs British)	1.31	0.65–2.66	0.447
Other/Mixed/Not stated (vs British)	1.22	0.70–2.13	0.490
Shoulder dystocia	3.55	1.94–6.47	<0.001
Birthweight ≥4,000 g	1.88	1.18–2.99	0.008
Mid-cavity forceps (vs low forceps)	1.90	0.99–3.65	0.055
Rotational forceps (vs low forceps)	1.69	0.48–5.91	0.412
Ventouse with rotation (vs low forceps)	0.29	0.07–1.24	0.095
Ventouse no rotation (vs low forceps)	0.38	0.22–0.66	<0.001
Spontaneous vaginal (vs low forceps)	0.31	0.18–0.55	<0.001
Maternal age ≥35 years	1.33	0.98–1.79	0.063
BMI ≥30 kg/m²	0.69	0.46–1.04	0.080
Episiotomy (yes vs no)	0.30	0.19–0.49	<0.001
Induced labour (vs spontaneous)	0.72	0.55–0.95	0.021

Complete-case multivariable logistic regression with operative vaginal delivery stratified by detailed mode.

n = 8,568; OASIS events = 245.

Subgroup counts: low forceps n=662 (40 OASIS); mid-cavity forceps n=141 (15 OASIS); rotational forceps n=36 (3 OASIS); ventouse with rotation n=113 (2 OASIS); ventouse no rotation n=912 (23 OASIS); SVD n=7,709 (187 OASIS).

Reference categories: multiparous; British ethnicity; low-cavity forceps; episiotomy not performed; birthweight <4,000 g; maternal age <35 years; BMI <30 kg/m²; spontaneous onset. Small subgroup estimates (in particular rotational forceps, n=36 with 3 OASIS events) should be interpreted with caution given the wide confidence intervals.

Supplementary Table S2. Per-variable missingness in the analytic cohort.

Variable	Missing (n)	Missing (%)
Maternal age	0	0
Booking BMI	284	2.96
Parity	0	0
Ethnicity	0	0
Shoulder dystocia	16	0.17
Mode of delivery	0	0
Episiotomy	716	7.47
Birthweight	4	0.04
Onset of labour	25	0.26
Diabetes	299	3.12
Infant sex	4	0.04

Analytic cohort n = 9,586. Variables with <8% missing data were handled with complete-case analysis in the primary model.
